# MicroRNA and Nonsense Transcripts as Putative Viral Evasion Mechanisms

**DOI:** 10.3389/fcimb.2019.00152

**Published:** 2019-05-08

**Authors:** Abhijeet A. Bakre, Ali Maleki, Ralph A. Tripp

**Affiliations:** ^1^Department of Infectious Diseases, College of Veterinary Medicine, University of Georgia, Athens, GA, United States; ^2^Department of Virology, Faculty of Medical Sciences, Tarbiat Modares University, Tehran, Iran

**Keywords:** RNA viruses, microRNA, miRNA, miR, seed sequence, mimics, sponges

## Abstract

Viral proteins encode numerous antiviral activities to modify the host immunity. In this article, we hypothesize that viral genomes and gene transcripts interfere with host gene expression using passive mechanisms to deregulate host microRNA (miRNA) activity. We postulate that various RNA viruses mimic or block binding between a host miRNA and its target transcript, a phenomenon mediated by the miRNA seed site at the 5′ end of miRNA. Virus-encoded miRNA seed sponges (vSSs) can potentially bind to host miRNA seed sites and prevent interaction with their native targets thereby relieving native miRNA suppression. In contrast, virus-encoded miRNA seed mimics (vSMs) may mediate considerable downregulation of host miRNA activity. We analyzed genomes from diverse RNA viruses for vSS and vSM signatures and found an abundance of these motifs indicating that this may be a mechanism of deceiving host immunity. Employing respiratory syncytial virus and measles virus as models, we reveal that regions surrounding vSS or vSM motifs have features characteristics of pre-miRNA templates and show that RSV viral transcripts are processed into small RNAs that may behave as vSS or vSM effectors. These data suggest that complex molecular interactions likely occur at the host-virus interface. Identifying the mechanisms in the network of interactions between the host and viral transcripts can help uncover ways to improve vaccine efficacy, therapeutics, and potentially mitigate the adverse events that may be associated with some vaccines.

## Introduction

Regulation of gene expression is a complex occurrence involving transcriptional and post-transcriptional mechanisms (Burgess, 2017). Translation of host mRNAs is regulated by small evolutionarily conserved small non-coding RNAs or miRNAs (Chen et al., 2017; Morales et al., 2017; Murashov, 2017) principally by sequence complementarity. Sequence complementarity is also fundamental to pathways such as clustered regularly interspaced short palindromic repeats (CRISPR) and RNA interference (RNAi) pathways (Panek et al., 2016; Kaikkonen and Adelman, 2018; Damas et al., 2019; Hussain et al., 2019) of which miRNAs have an integral function. Host miRNA genes can be intronic, intergenic, or independent transcription units typically processed by RNA polymerase II. Initial transcription produces primary miRNA transcripts (pri-miRNA) which are processed by a nuclear microprocessor complex to produce pre-miRNAs (Bartel, 2004; Finnegan and Pasquinelli, 2013; Ha and Kim, 2014). Pre-miRNAs are exported out into the cytoplasm and further processed into a 18–25nt long dsRNA by class III RNAse enzyme Dicer to form the mature mRNA duplex. One strand of the duplex is thermodynamically stable and referred to as the guide strand while the other strand is the passenger strand. The mature guide strand at the 5' end encodes a 6 nt ‘miRNA seed’ that is complementary to a miRNA recognition element (MRE) in target transcripts (Bartel, 2004; Finnegan and Pasquinelli, 2013; Ha and Kim, 2014). Sequence dependent pairing of the seed site with MRE can lead to either mRNA decay or blocked translation (Fabian et al., 2010). Each miRNA can target several genes although the stoichiometry of this interaction is inadequately defined (Weill et al., 2015). Non-seed-mediated miRNA regulation has also been recognized (Fabian et al., 2010; Ghosal et al., 2014; Li et al., 2014; Kumari et al., 2016), is an alternate mechanism of miRNA function (Cloonan, 2015), and multiple miRNAs may regulate a single gene (Bartel, 2009; Friedman et al., 2009; Fabian et al., 2010). There are several features that may contribute to function as different motifs may be able to interact among viral and host transcripts given each position can have one of the four nucleotides (A/U/G/C) and the six nt in the seed site can be promiscuous and bind to many targets (Friedman et al., 2009). Host gene miRNA regulation is well established and has been demonstrated to modulate ~60% of the human transcriptome (Friedman et al., 2009; Dong and Lou, 2012; Hashimoto et al., 2013; Jia et al., 2014). It is well known that miRNAs regulate cell physiology during normal homeostasis as well as during disease states (Karnati et al., 2015; Alipoor et al., 2016; Vishnoi and Rani, 2017; Olejniczak et al., 2018). Importantly, differential expression of miRNAs has been used as biomarkers for diagnosis, treatment and prognosis, and miRNA expression is modified in response to viral infection (Sullivan and Ganem, 2005; Piedade and Azevedo-Pereira, 2016; Auvinen, 2017; Trobaugh and Klimstra, 2017).

DNA viruses typically replicate with a high fidelity and encode their own miRNAs (Klinke et al., 2014; Flor and Blom, 2016; Albanese et al., 2017; Qin et al., 2017) to regulate virus and host gene expression. In contrast, RNA viruses lack replicative fidelity and arise in the host following infection as swarms of quasispecies (Steinhauer et al., 1992). The quasispecies generally have poor replicative fitness. RNA virus replication and gene transcription are catalyzed by RNA-dependent RNA polymerase (RdRP) which is error-prone (Perez-Rodriguez et al., 2016). It is not understood what proportion of quasispecies are translated during infection, though non-canonical start codons can be used during RNA viral protein synthesis (Firth and Brierley, 2012). Thus, quasispecies can contribute to antigenic diversity via non-canonical translation of alternative viral proteins, and inhibition of these processes can improve viral yield during vaccine production via molecular breeding, increase vaccine safety and stability and reduce potential adverse events (Perez-Rodriguez et al., 2016). While a lack of exonuclease proof reading activity occurs in the RdRP, a feature explaining how low fidelity arises, it remains unclear why RNA viruses have and maintain low fidelity RdRP. It is possible that low RdRP fidelity facilitates virus replication (Hopfield, 1974; Back et al., 1996) by allowing RNA viruses to escape situations where unfit mutations predominate fitness leading to species collapse and attenuation (Lauring and Andino, 2010).

In this article, we speculate that RNA viruses interfere with the host RNAi machinery that regulates both foreign and endogenous gene expression by miRNAs. We postulate that RNA viral genomes and gene transcripts encode motifs that can either mimic or block native miRNA activity through sequence homology or complementarity. We analyzed the genomes of several RNA virus families (Supplementary Tables 1, 2) and identified vSMs that potentially may mimic miRNA seed activity and thus increase native miRNA repression of host antiviral pathways. We also identified several viral seed sponges that can potentially block host miRNAs and relieve native miRNA suppression of pro-viral host genes. We focused our analysis on the *Paramyxoviridae* family of RNA viruses owing to their impact on human and animal health. Paramyxoviruses have negative sense, non-segmented, single-stranded RNA genomes that are transcribed in a gradient leading to a differential abundance of viral transcripts with all steps in the viral life cycle occurring in the cytosol where host miRNAs also regulate gene expression. Paramyxoviruses are classified into two subfamilies *Paramyxovirinae* (e.g., Avulavirus, Henipavirus, Morbilivirus, Respirovirus, and Rubulavirus genera) and *Pneumovirinae* (e.g., Pneumovirus and Metapneumovirus genera) (Aguilar and Lee, 2011; Amarasinghe et al., 2017; Rima et al., 2017). Paramyxovirinae members causing morbidity and mortality include measles (MV), Mumps (MuV), Hendravirus (HV), Nipah virus (NiV), and the Pneumoviruses, i.e., respiratory syncytial virus (RSV) and human metapneumovirus (hMPV).

In this article, we propose that quasispecies enable RNA viruses to modulate host gene expression by regulating miRNA function via sequence complementarity or identity with the miRNA seed sites. We also suppose that vSM function to increase native miRNA-based suppression, while vSS inhibit native miRNA activity and increase host gene expression to the advantage of the virus. Preliminary analysis has identified a number of vSM or vSS in several Paramyxovirus genomes. For example, for RSV the regions that neighbor potential vSS or vSM are predicted to form stable stem loop structures that are typically substrates for nuclear and cytosolic RNAses of the RNAi pathway (Cai et al., 2004; Ritchie et al., 2007; Shu et al., 2007; Kurihara and Watanabe, 2010). These findings suggest that these regions in the viral genome can be templates for cytosolic Dicer activity. We have confirmed that during RSV infection gene transcripts are processed into sncRNAs and have identified viral transcripts that harbor vSS or vSM using next generation sequencing (NGS). Genomic analyses show that these motifs are more abundant in genes that have known or predicted immunomodulatory function or are involved in viral replication/transcription. These data suggest that interactions with host miRNAs may be part of a mechanism to modulate miRNA-mediated host regulatory pathways and regulate viral gene expression. These findings have important implications for better understanding of host-virus interaction as well as rational vaccine design strategies.

## Materials and Methods

### Viruses and Cell Culture

Mycoplasma-free virus stocks of wild type RSV strain A2 were expanded in Vero cells (ATCC CCL81) and maintained in DMEM (Hyclone, Salt Lake City, Utah USA) supplemented with 5% heat-inactivated fetal bovine serum (Hyclone, USA) as previously described (Oshansky et al., 2009). A549 cells (ATCC-CCL185) grown in DMEM supplemented with 5% serum as above were used for all infections. A549 cells were infected at a multiplicity of infection (MOI) of 1.0 as previously described (Oshansky et al., 2009). SHSY5Y cells were maintained in DMEM with 10% heat inactivated FBS.

### Nucleotide Sequence Analysis

Complete genome sequences for RSV, MV, HMPV, MuPV, NDV, HV, NiV, MuV were from the National Center for Biotechnology Information (NCBI). Accession numbers for all sequences analyzed are given in Supplementary Table 1. A local database of human mature miRNA sequences version 21.0 was constructed locally in BioEdit. Viral sequences were analyzed using BLASTN (Altschul et al., 1990; Altschul and Gish, 1996; Altschul and Pop, 2017) against this local database using the parameters Expect value (E) = 10, matrix (M) = BLOSUM62, Low complexity Repeat masking = OFF and output = Tabular. CSV files were imported into Microsoft Excel 2010 and filtered to identify hits where miRNA start or miRNA end was ≤3. Hits in the same orientation as the miRNA (5′-3′) were designated vSMs while vSSs were in anti-sense orientation.

### Structure Prediction of RSV vSMs (mimics) and vSSs (sponges)

Nucleotide sequences (100 nt) flanking each predicted vSM or vSS for RSV were extracted from parental genomic sequence and analyzed by miRNAfold (Sullivan and Ganem, 2005) or RNA structure (Xu and Mathews, 2016). Structures were visualized with VARNA GUI (Hashimoto et al., 2013). Pre-miRNA sequences were used as controls in prediction. Hybridization stability was calculated using RNA hybrid (Jia et al., 2014).

### Analysis of Small RNA Processing

Total RNA from mock-treated or RSV A2-infected (MOI = 1.0) Vero cells was isolated and fractionated using RNAzol RT according to the manufacturer protocol (MRCgene, Ohio). Fractionated small RNA was polyadenylated and then reverse transcribed with Protoscript II (New England Biolabs, MA) RSV G- and L-specific reverse oligomers followed by PCR using gene specific forward and reverse oligomers in a reaction containing 10 uM final primer concentration as per manufacturer's recommendations. PCR amplicons were resolved on a denaturing 12% PAGE gel in 1X TBE buffer and stained with SYBR Gold. Sequences for RSV G and L oligomers are provided in Supplemental Methods.

### Next Generation Sequencing (NGS)

Type II respiratory epithelial (A549) cells or neuronal (SHSY5Y) cells were mock-treated or infected with RSV A2 (MOI = 1.0) for 24 h. Total RNA was isolated using RNAzol RT (MRCgene, Ohio) per the manufacturer protocol and size fractionated. Small RNA was quantified using Qubit broad range RNA kit (Invitrogen, USA). Size fractionated small RNA was ligated to proprietary Illumina adaptors using T4 RNA Ligase deletion mutant (Epicenter, USA). Adaptor ligated RNA was reverse transcribed and amplified for limited number of cycles during which index barcodes were attached to each cDNA pool. cDNA libraries for each sample at the end of the incubation temperature were again analyzed on a Tapestation 2200, quantified and then pooled at equimolar ratios as recommended by manufacturer. Pooled cDNAs were denatured using freshly prepared 0.2 N NaOH at room temperature for 3 min and then mixed with hybridization buffer. Diluted libraries at 20 pM concentration were loaded onto Illumina MiSeq and run to generate fastq files as per Illumina MiSeq protocol. Sequencing reads obtained had quality scores >30, were trimmed of adaptors, and were then analyzed by BLAST against RSV A2 genome to determine virus specific transcripts and determine gene location for these transcripts.

## Results

### *Paramyxovirus* Transcripts Mimic or Bind to Host miRNAs by Seed Sequence

**Figure 1:**
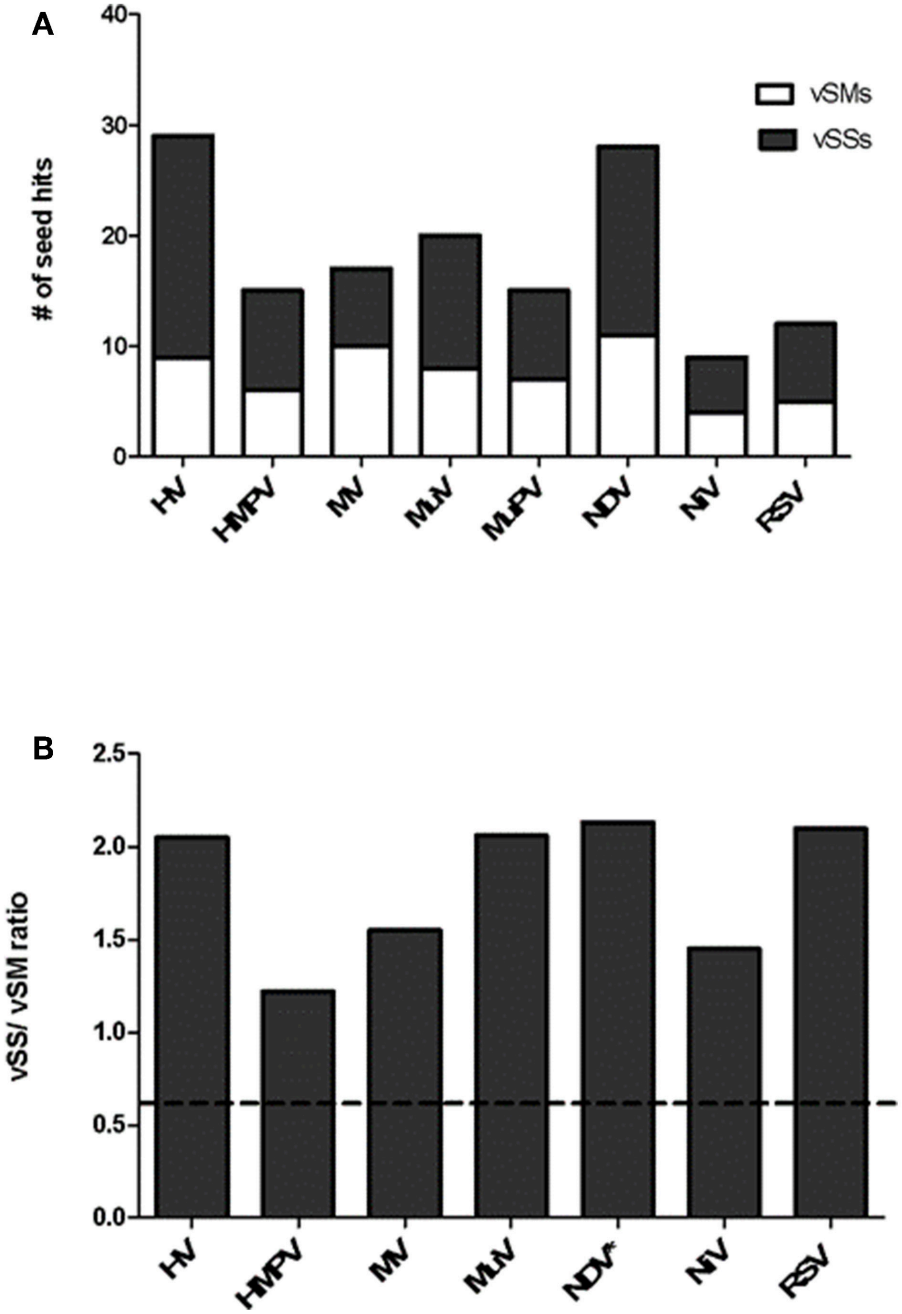
Distribution of vSMs and vSSs across genera of the Paramyxoviridae family. **(A)** Viral sequences identical to host miRNA seed site were designated vSMs (open bars) while those complementary to miRNA seed sites were designated as vSSs (filled bars). **(B)** Ratio of vSS/vSM across genera is shown. Dotted line represents ratio equal to 1.0. HV, hendravirus; HMPV, human metapneumovirus; MV, measles virus; MuV, mumps virus; NDV, New Castle's disease virus; NiV, nipah virus; RSV, respiratory syncytial virus.

*Paramyxovirus* genome replication, gene transcription, and translation occur in the cytoplasm, which is the primary site of host post-transcriptional gene regulation by miRNAs. To identify the sequence motifs in *Paramyxovirus* genomes that could mimic or inhibit host miRNA function based on seed sequence homology/complementarity, RSV, HMPV, MuPV, NDV, HV, NiV, MV, and MuV genomes were analyzed by basic local alignment search tool (BLAST) (Altschul et al., 1990) against a database of either human mature miRNAs or cognate species using criteria optimized for finding short matches (matrix = BLOSUM 62, Expect value = 10). Hits were filtered to identify matches in the miRNA seed site that were either in the sense (+/+) or antisense (±) orientation relative to the miRNA. Sense orientation hits were designated as vSMs (Table 2), while antisense hits were designated vSSs (Table 3). Across genera, we identified 85 vSSs compared to 60 vSMs for the type strains (Figure 1A). The ratio of the number of vSSs to vSMs was typically in the 1.5–2.0 + range for most genera except HMPV where they were in approximately equal numbers (Figure 1B). The ratio of vSS/vSM was determined to determine if vSMs vs. vSSs were selected for or against during evolution. As noted in the Introduction, vSMs are expected to enhance native miRNA mediated suppression while vSSs are expected to bind to miRNAs and relieve their repressor effect. A high vSM/vSS ratio would indicate that mimicking host miRNA activity could be a predominant mechanisms of modulating host miRNA activity. Alternatively, a low vSM/vSS ratio might indicate that inhibiting miRNA activity would be a more conserved mode of regulating miRNA function. Most vSMs or vSSs were located in the L gene across the *Paramyxovirus* genera followed by F protein and G protein genes. Details of vSMs and vSSs in are discussed below.

### Respiratory Syncytial Virus

**Figure 2:**
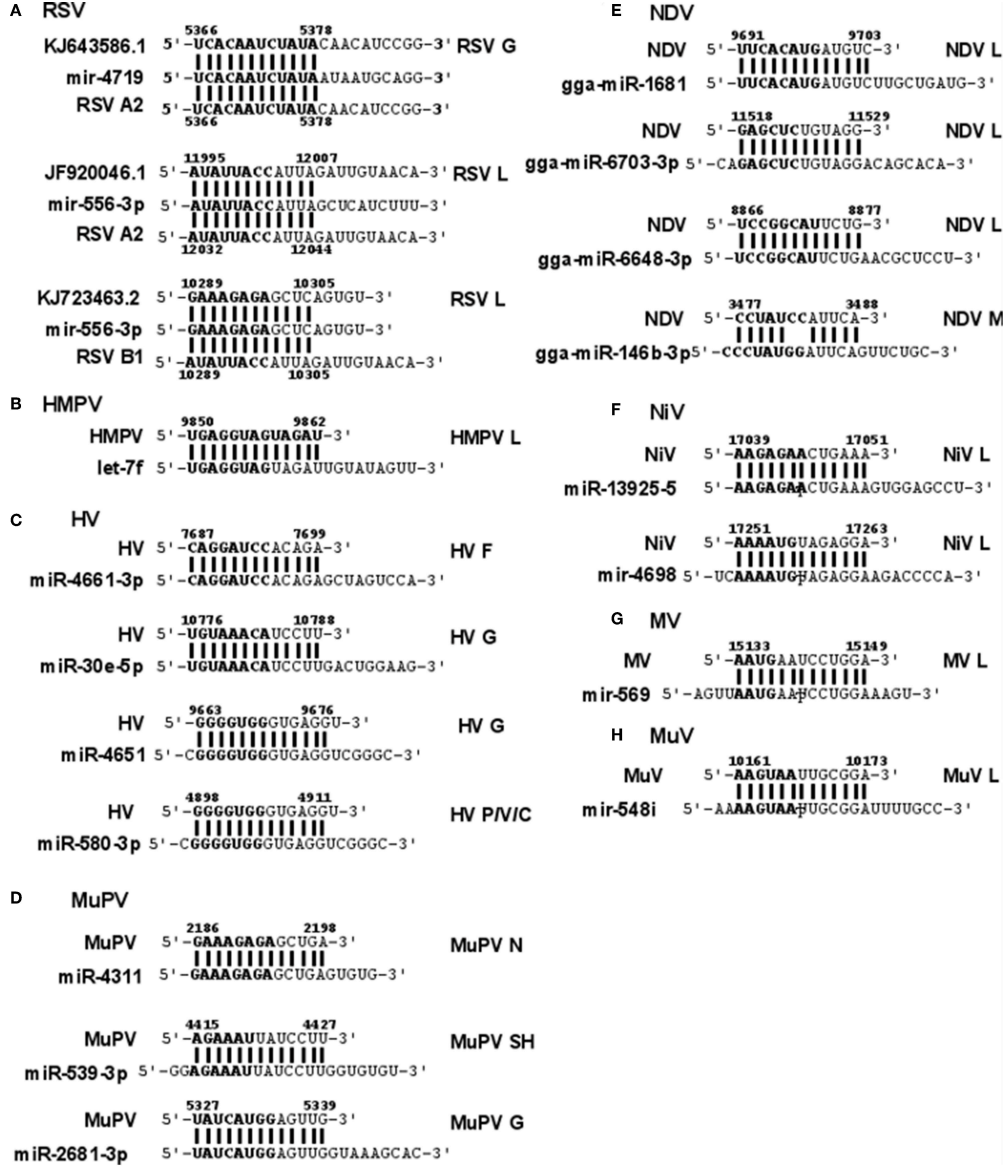
Sequence alignments of miRNA vSMs encoded by different paramyxoviruses. Alignments show identity between host miRNA seed sites and the corresponding vSM across *Paramyxoviruses*. Nucleotide numbers indicated viral genome coordinates while labels on the right indicate encoding viral gene. miRNA seed sequence is indicated in bold. Straight lines indicate perfect identity.

**Figure 3:**
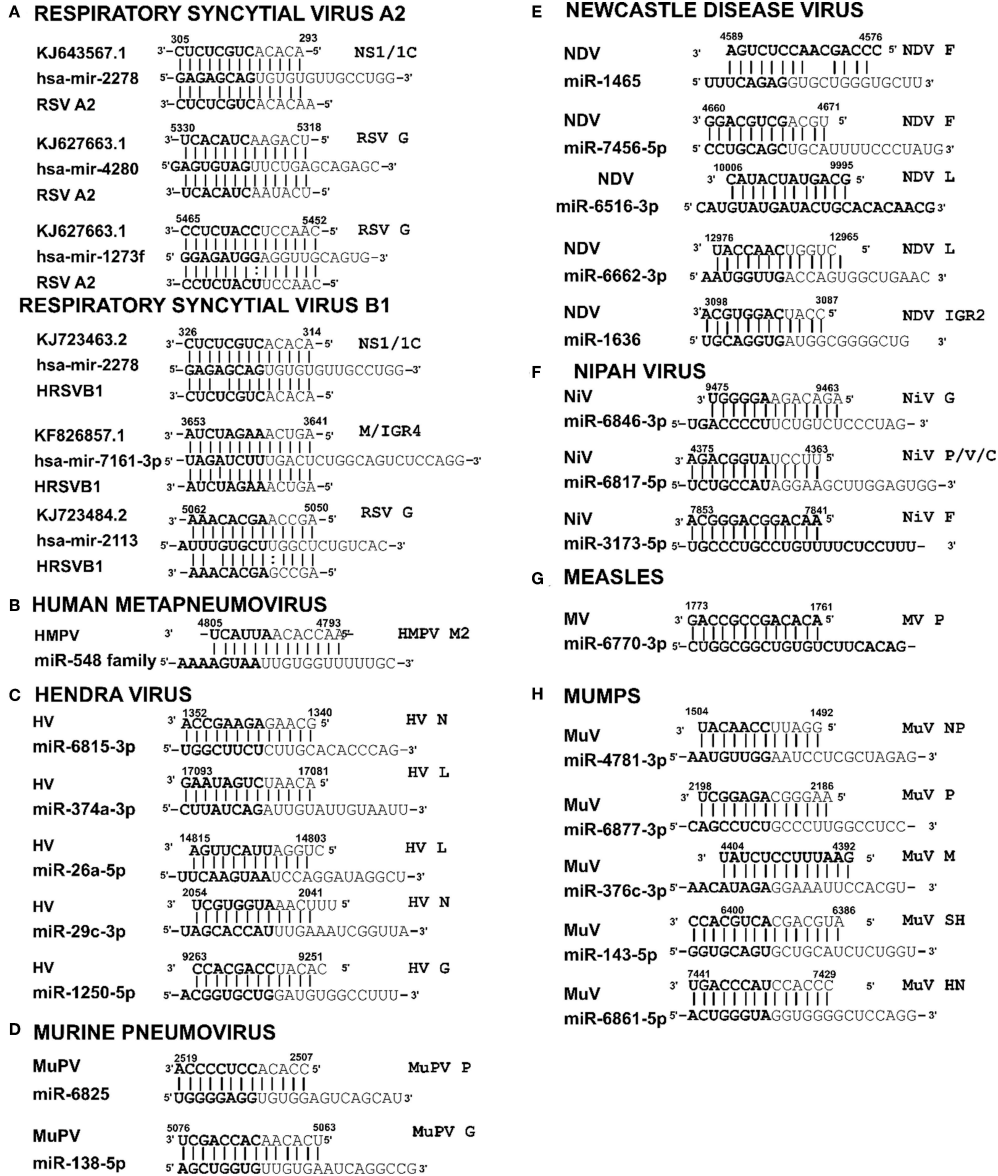
Sequence alignments of miRNA vSSs encoded by different paramyxoviruses. Alignments show complementarity between host miRNA seed site and the corresponding vSS across *Paramyxoviruses*. Nucleotide numbers indicated viral genome coordinates while labels on the right indicate encoding viral gene. miRNA seed sequence is indicated in bold. Straight lines indicate Watson-Crick base pairing (AU/GC) while colon indicates a non-Watson-Crick base pair (GU).

RSV is grouped as A and B strains based on the diversity in the G protein (Johnson and Graham, 2004; Papenburg and Boivin, 2010; Pangesti et al., 2018). For strain A2 (GenBank accession number M74568), we did not identify any vSM or vSS while for strain B1 (GenBank accession number AF013254), one vSM was identified in the L gene. Analysis of clade A (326 sequences identified) and clade B (60 sequence strains) (accession numbers in Supplementary Table 1) identified two vSMs, one mapping to miR-4719 in the G gene and the other mapping to miR-556-3p in L gene (Figure 2A). Strains that encoded vSM-4719 did not encode vSM-556-3p and vice-versa. For B1, the single vSM corresponding to miR-4311 mapped to the L gene. The viral gene segments that encoded vSSs included miR-2278 in the NS1/1c gene and miR-4280 and miR-1273f in the G gene (Figure 3A).

### Human Metapneumovirus

HMPV, like RSV, is grouped as A and B strains based on G gene diversity (Kahn, 2006; Papenburg and Boivin, 2010; Schuster and Williams, 2014). A single vSM for miRNA let-7f was located in the L gene (Figure 2B), and a single vSS complementary to multiple members of the miR-548 family was found in the M2 gene (Figure 3B).

### Hendravirus

Analysis of the HV genome identified three major vSMs for miR-4661-3p, miR-30e-5p, and miR-580-3p in F, G, and P genes (Figure 2C). vSSs for miR-6815-3p and miR-29c-3p in the N gene, miR-374a-5p, and miR-26a-5p in the L gene, and miR-1250-5p in the G gene were identified (Figure 3C).

### Murine Pneumovirus

Analysis of MuPV genome identified several conserved vSMs containing seed sequence of miR-4311 in the N gene, miR-539-5p in the SH gene, and miR-2681-3p in the G gene (Figure 2D), as well as vSSs complementary to miR-6825 and miR-138-5p in the P and G genes, respectively (Figure 3D).

### New Castle Disease Virus

Avian Paramyxovirus-1 (APMV-1) is the etiological agent of NDV. Analysis of the APMV-1 genome identified four highly conserved vSMs for miR-1681, miR-6703-3p, and miR-6648-3p in the L gene and miR-146b-3p in the M gene (Figure 2E). The genome also encoded two vSSs each for miR-1465, miR-7456-5p in the F gene, and miR-6516-3p and miR-6662-3p in the L gene, and miR-1636 in the intergenic region (Figure 3E). APMC-1 encoded the highest number of vSMs and vSSs among all paramyxoviruses.

### Nipah Virus

Analysis of NiV genomes identified two vSMs for miR-3925-5p and miR-4698 in the L gene (Figure 2F). The genomes also encoded vSSs against miR6846-3p in the G gene, miR-6817-5p in the P gene and miR-3173-5p in the F gene (Figure 3F).

### Measles Virus

The MV genome had a vSM for miR-569 in the L gene (Figure 2G), and a vSS for miR-6770-3p in the P gene (Figure 3G).

### Mumps Virus

MuV genome has a vSM in the L gene for mir-548i (Figure 2H). The genome also encodes five vSSs against miR-4781-3p in the NP gene, miR-6877-3p in the P gene, miR-376c-3p in the M gene, miR-143-5p in the SH gene, and miR-6861-5p in the HN gene (Figure 3H).

These comparative analyses of *Paramyxoviruses* revealed an enrichment of vSSs relative to vSMs and localization of vSM sequences in L or G genes. It is not clear if these miRNA sequences evolved as an adaptation to a host response or are an outcome of low polymerase (L gene) fidelity. The conservation of these sequences in the circulating viral strains of multiple paramyxoviruses suggests that they may facilitate virus replication possible by contributing to immune evasion.

### Viral RNA Is Processed to Smaller Transcripts During Infection

**Figure 4:**
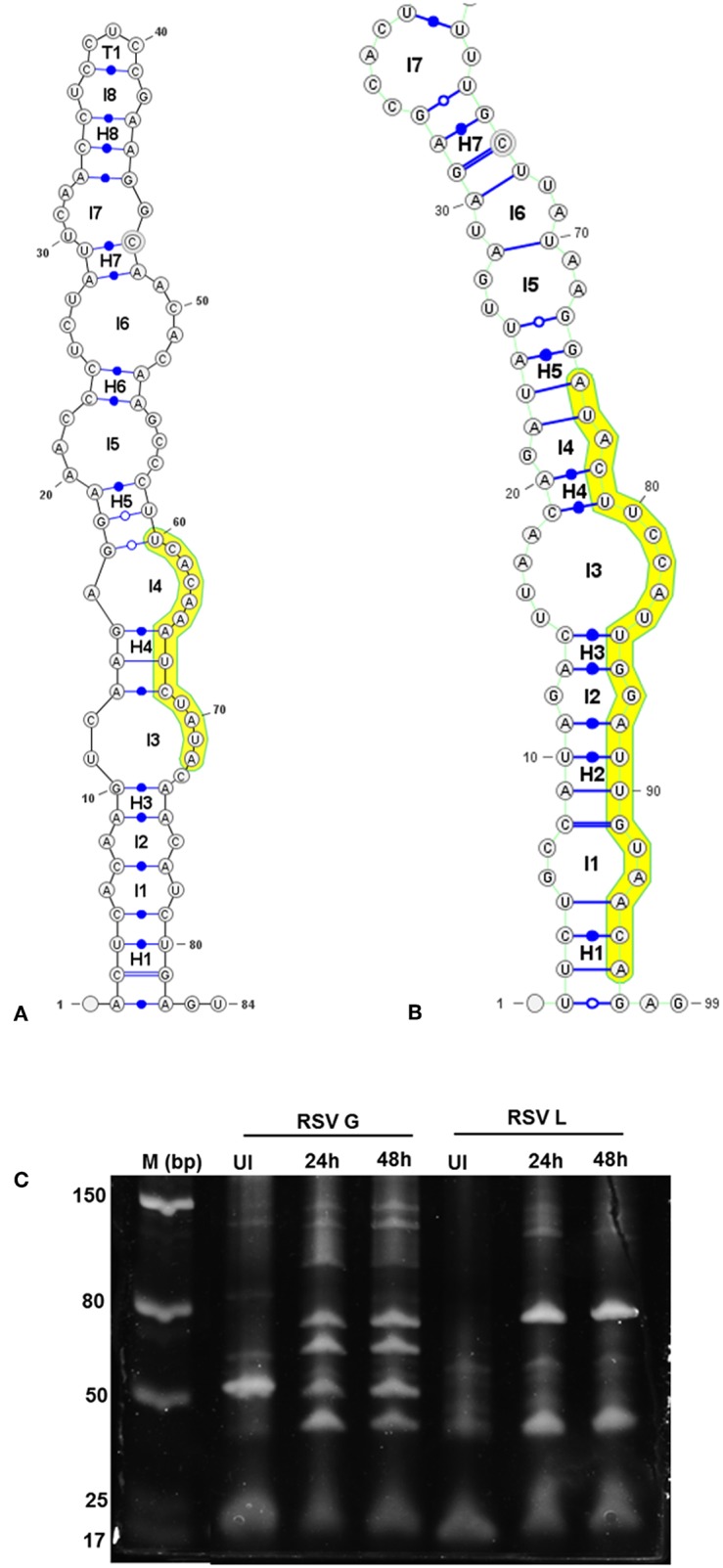
Viral genomic regions form characteristic stem- loop folds in vSM and vSS encoding regions and are processed into smaller transcripts. Secondary structures of a 100 nt sequence flanking vSM-4719 **(A)** and−556-3p **(B)** were predicted using RNAfold and drawn using VARNA. Highlighted region corresponds to the vSM. Internal and terminal loops are prefixed with L and T, respectively. Helices are prefixed with H and the 5′ and 3′ ends of the molecule are as indicated. **(C)** Size-fractionated small RNA from mock or RSV A2 infected (24 h or 48 h pi) Vero cells was reverse transcribed using RSV G- and L-specific reverse primers. Amplicons obtained using a G/L specific primer pair were electrophoresed on a 12% PAGE gel alongside a molecular ladder and stained with SYBR gold.

Stem-loop structures are a feature of miRNA templates and are recognized by both nuclear and cytosolic class III RNAses (Fabian et al., 2010; Weill et al., 2015). Several studies suggested RSV genome/antigenome might not fold to form secondary structures due to N protein encapsidation (Ghosal et al., 2014; Kumari et al., 2016). However, recent data has shown that RNA probes can bind to RSV genomic RNA (Bartel, 2009; Li et al., 2014; Cloonan, 2015) support the hypothesis that the RSV genome/antigenome is exposed during infection and may be able to form secondary structural motifs that can act as templates for Dicer, a class III cytosolic RNAse (Friedman et al., 2009). There is no published data to indicate that viral transcripts are protein encapsidated in the cytosol during infection. We analyzed genomic sequences near predicted vSMs and vSSs, and RNA secondary structure predictions indicated the potential of these regions to fold into stable stem loop structures similar to pre-miRNA transcripts (Figures 4A,B). Randomization of the sequence disrupted the structure. Stability of interaction between miRNAs and vSSs were computationally determined with RNAhybrid, a tool for finding the minimum free energy hybridization of a long and a short RNA to predict miRNA targets (Jia et al., 2014). Interactions between the predicted vSS and complementary miRNA are very stable as evident from the low mean free energy of the hybrid (Table 1). To establish if the RSV transcripts are processed into small RNAs during infection, Vero cells were infected with RSV A2 (MOI = 5.0) for 24 or 48 h, and size-fractionated small RNA was reverse transcribed using G- or L- specific oligonucleotides and amplified using G- or L-specific forward and reverse oligomers. MOI of 1.0 was used to infect A549 or SHY5Y cells with RSV or MV followed by small RNA isolation at 24hpi and NGS sequencing. MOI of 5.0 was used to infect Vero cells for RT-PCR analysis of small RNA products. The different MOIs were used because of the different sensitivities of the assay methods. With NGS, we expected to observe even small differences owing to the extremely high sensitivity of the method. In contrast, for Vero cells, RSV infection produces little to no cytopathic effect at 24–48 hpi, thus necessitating higher MOI infection. Electropherograms clearly showed bands of ~40, 50, 70, and 80 bp for the G gene, and 40 and 80 bp for the L gene along with several bands in the ~120 bp size range that are similar to host pre-miRNA sizes (Figure 4C). These data support the hypothesis that vSM- and vSS-encoding regions of the genome are folded and processed into smaller transcripts potentially via cellular RNAi machinery. Experimental validation of predicted structures is out of the scope of this hypothesis article.

**Table 1:** Binding energy calculations for vSSs per RNA duplex.

**Virus**	**vSS**	**miRNA**	**MFE (Kcal/mol)**
RSV A2	NS1-2278	miR-2278	−32.4
	G-4280	miR-4280	−25.2
	G-1273f	miR-1273f	−29.7
RSV B1	NS1-2278	miR-2278	−31.9
	M/IGR4-7161-3p	miR-7161-3p	−34.0
	G-2113	miR-2113	−29.0

*Stability of vSS miRNA was determined using RNAhybrid. Mean free energy (MFE) denotes stability of interaction. NS1, Non-structural gene-1; G, RSV G gene; M, Matrix gene. Number indicate the potential miRNA target*.

**Table 2:** vSMs in *Paramyxovirus* genomes.

**Genera**	**vSMs**
RSV A	miR-4719 > miR-556-3p > miR-4801 > miR-8074 > miR-3613-3p > miR-1253 > miR-3618 > miR-182-5p > miR-8485 > miR-4770
RSV B	miR-4311 > miR-6780a-5p > **let-7b-3p** > miR-499a-3p
HMPV A	**miR-4662a-5p** **>** **miR-20b-5p** **>** let-7f-5p > miR-5194 > miR-548u > **miR-1267** > **miR-3925-5p** > miR-3148 > miR-4799-5p > **miR-6884-5p** > miR-335-5p
HMPV B	**miR-6884-5p** > miR-592 > **miR-1267** > **miR-20b-5p**
NDV	miR-6876-5p > miR-1306-5p > miR-1264 > miR-4280 > miR-6888-3p > miR-6855-3p > miR-338-3p > miR-548aq-5p > miR-6875-5p > miR-8069 > miR-3925-3p > miR-1249-5p > miR-892b > miR-4656 > miR-4679
HV	miR-4661-3p > miR-4651 > miR-30e-5p > miR-6734-5p
NiV	**miR-3925-5p** **>** miR-4698 > miR-4693-3p
MV	miR-374a-3p > miR-5571-5p > miR-3942-3p > **miR-4662a-5p** **>** miR-5688 > miR-6861-3p > miR-4275 > miR-3617-3p > miR-4273 > miR-569 > miR-3202 > miR-1297 > miR-26b-5p > miR-3934-3p > miR-1255b-5p > miR-6755-3p > miR-1469 > miR-324-3p > miR-548ah-5p > miR-593-3p > miR-4518 > miR-6803-3p > miR-4690-5p > miR-4466 > miR-210-3p > miR-2909 > miR-3909 > miR-4505 > **let-7b-3p** > miR-2276-5p > miR-4420 > miR-4498 > miR-6075 > miR-2276-3p > miR-4486 > miR-6793-3p
MuV	miR-6861-5p > miR-6877-3p > miR-143-5p > miR-4433a-5p > miR-1236-3p > miR-4781-3p > miR-219a-2-3p > miR-1233-5p > miR-4645-5p > miR-3131 > miR-6881-5p > miR-4269 > miR-7152-3p > miR-4286 > miR-4323

*vSMs in bold are conserved across two or more viruses. vSM hits are listed in order of their decreasing abundance among viral genomes. RSV, respiratory syncytial virus; hMPV, human metapneumovirus; NDV, New Castle disease virus; HV, Hendravirus; NiV, Nipah virus; MV, Measles virus; MuV, Mumps virus*.

**Table 3:** vSSs in Paramyxovirus genomes.

**Genera**	**vSSs**
RSV A	**miR-2278** > miR-4280 > miR-1273f > miR-4753-3p > miR-592 > miR-4639-3p
RSV B	**miR-2278** **>** **miR-7161-3p** > miR-2113 > miR-7151-5p > miR-8068 > miR-369-3p
HMPV A	**miR-8063** **>** **miR-323b-5p** **>** **miR-6831-5p** **>** **miR-337-5p** **>** miR-5584-3p > **miR-494-5p** **>** **miR-3678-3p**
HMPV B	**miR-891a-3p** **>** **miR-6831-5p** **>** **miR-323b-5p** **>** **miR-337-5p** **>** **miR-8063** **>** **miR-3678-3p** > miR-6730-3p
NDV	miR-219a-1-3p > miR-3165 > miR-1257 > miR-758-5p > **miR-7161-3p** > miR-7844-5p > miR-6868-3p
HV	miR-374a-3p > miR-1250-5p > miR-6815-3p > miR-29b-3p > miR-29c-3p > miR-26a-5p > miR-6740-3p
NiV	miR-6817-5p > miR-6846-3p > miR-3173-5p > miR-1205 > miR-2054
MV	miR-6770-3p > miR-4455 > miR-514b-3p > miR-6506-5p > miR-1183 > miR-6841-3p > miR-4714-5p
MuV	miR-6861-5p > miR-6877-3p > miR-143-5p > miR-4433a-5p > miR-1236-3p > miR-4781-3p > miR-219a-2-3p

*vSSs in bold are conserved across two or more viruses. vSSs identified Paramyxovirus genomes. vSMs in bold are conserved across two or more viruses. vSS hits are listed in order of their decreasing abundance amongst viral genomes. RSV, respiratory syncytial virus; hMPV, human metapneumovirus; NDV, New Castle disease virus; HV, hendravirus; NiV, nipah virus; MV, measles virus; MuV, mumps virus*.

### Small Viral RNA Transcripts Encode vSMs and vSSs

**Figure 5:**
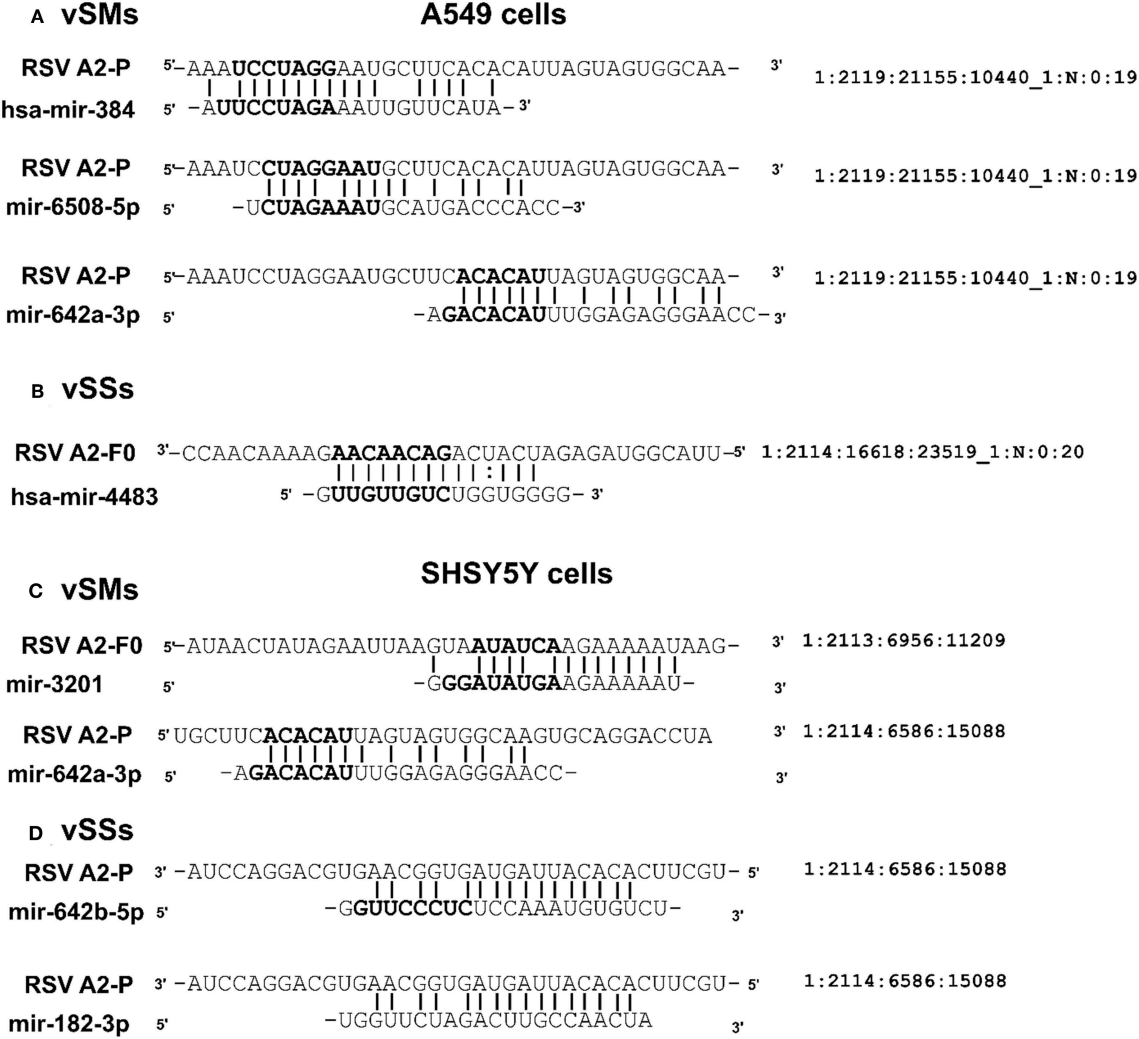
Small RNA from RSV-infected respiratory epithelial (A549) **(A,B)** or neuronal (SHSY5Y) **(C,D)** cells were sequenced on an Illumina MiSeq. Alignments show RSV vSMs or vSSs in bold.

The results show that RSV infection of Vero cells, which lack a functional IFN α/β locus (Karnati et al., 2015; Alipoor et al., 2016), produces small RNAs from the G and L genes (Figure 4C). We also analyzed small RNAs transcriptomes of mock-treated, RSV-infected A549 cells, or RSV-infected neuronal SHSY5Y cells by NGS to identify if these transcripts encoded vSMs and vSSs. Transcripts mapping to viral genome over their entire length (36 nts) were also compared to mature human miRNAs to identify vSMs and vSSs. In RSV-infected A549 cells, we identified viral transcripts that encoded three overlapping full-length vSMs (miR-384, miR-6508-5p, and miR-642a-3p) and others that were perfectly complementary to miR-4483 (Figure 5A). Viral transcripts from RSV infected SHSY5Y cells encoded a near full length miR-3201 or miR-642a-3p transcript and vSSs against miR-642b-5p and miR-182-3p (Figure 5C). Many of transcripts encoded only vSMs or vSSs in contrast to the transcripts noted above which encoded nearly full-length host miRNAs (Figures 5B,D). These findings support our hypothesis that *Paramyxovirus* gene transcripts may modulate host miRNA activity via seed match or complementarity. While host encoded competing endogenous RNA are known, this is the first such analysis done for *Paramyxovirus* genomes thereby opening new areas of investigation.

## Discussion

The low replicative fidelity of RdRp of *Paramyxoviruses* appears to be an evolutionary strategy since it allows for quasispecies generation, favors emergence of variants that can escape immune pressure, and may allow the virus to modulate host responses and gene expression via regulation of miRNA function. Tempering host gene expression is important for viral replication, and viruses have evolved several means to counter host responses to virus infection using mechanisms including shutdown of host transcription, translation, and modification of sncRNA and miRNA expression (Vishnoi and Rani, 2017; Olejniczak et al., 2018).

**Figure 6:**
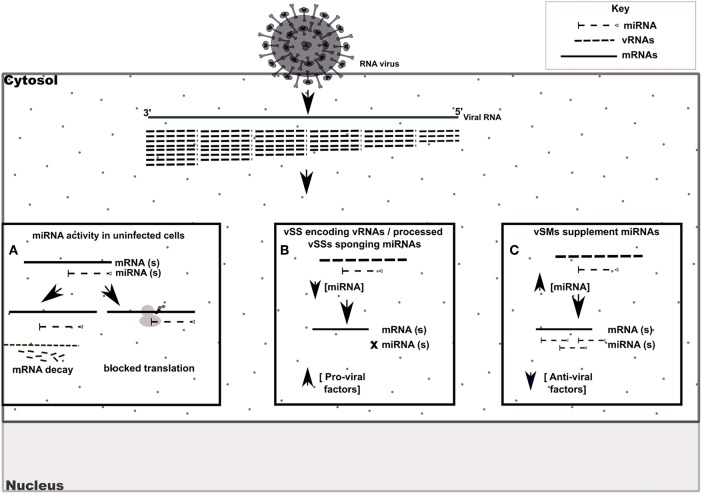
Model of vSM / vSS modulation of host responses during infection. Host mRNA are depicted as solid lines, microRNAs are depicted as dashed lines with end caps while viral transcripts are depicted as dashed lines. **(A)** Normal post-transcriptional miRNA regulation of gene expression. Degree of miRNA complementarity determines if gene knockdown proceeds via mRNA decay/translation following miRNA binding to target transcript in the RISC complex. **(B)** Viral RNAs encoding vSSs/processed vSSs bind to host miRNAs and prevent native suppression of pro-viral factors. **(C)** Viral RNAs encoding vSMs can supplement host miRNA activity and suppress anti-viral responses.

RIG-I and MDA-5 are prototypical pattern recognition receptors that detect and respond to presence of 5′ triphosphorylated and double-stranded RNA, respectively, during RNA virus infection (Piedade and Azevedo-Pereira, 2016; Auvinen, 2017). These receptors trigger a signaling cascade that culminates in establishment of an antiviral state in the infected and neighboring cells (Piedade and Azevedo-Pereira, 2016). Viruses avoid host responses to replicate and moderate these responses to facilitate replication. In addition to modifying the antiviral response, we propose that quasispecies generation during RNA virus replication helps regulate host gene expression by modulation of host miRNA function and activity. We speculate that RNA viral quasispecies produce a cloud of molecular signatures that mimic or inhibit host miRNA activity via sequence complementarity and alter the expression and function of several host pathways during infection. Our data suggest that vSMs and vSSs encoded by *Paramyxovirus* genomes and transcripts aid quasispecies generation by modulating various host pathways (Table 4) (Figure 6). It is likely that the generation of quasispecies is dynamic and adapts to host cell pressure. Thus, viral transcripts can mutate to produce vSMs or vSSs that modulate different miRNAs in different cell types as well as carry one or more frameshift mutations to produce alternative proteins/peptides that provoke modified or altered responses as these data suggest where viral transcripts can potentially bind to or mimic host miRNAs perturbing their expression and diverting host resources.

**Table 4:** miRs targeted by vSMs and vSSs for HMPV or RSV strain A or B were analyzed by DIANA miRPath server.

	**# of vSMs**			**# of vSSs**		
**Pathways deregulated by virus infection**	**RSV A**	**RSV B**	**Total**	**RSV A**	**RSV B**	**Total**
Prostate cancer	1	1	2	1	1	2
Axon guidance	1	1	2		1	1
**PI3K-Akt signaling pathway**	1	1	2	1	1	2
Chronic myeloid leukemia	1	1	2		1	1
Non-small cell lung cancer	1		1	1	1	2
Transcriptional mis-regulation in cancer	1	1	2	1		1
Endometrial cancer	1		1	1	1	2
T cell receptor signaling pathway	1	1	2		1	1
Focal adhesion	1	1	2			0
**Neurotrophin signaling pathway**	1		1		1	1
Vasopressin-regulated water reabsorption	1		1		2	2
ErbB signaling pathway	1		1		1	1
Regulation of actin cytoskeleton	1		1			0
Prion diseases	1	1	2			0
Glioma	1		1	1		1
Endocytosis	1	1	2			0
**Wnt signaling pathway**	1	1	2			0
Colorectal cancer	1	1	2			0
Ubiquitin mediated proteolysis	1	1	2			0
Dopaminergic synapse	1	1	2			0
**MAPK signaling pathway**	1	1	2			0
Renal cell carcinoma	1		1		1	1
Melanogenesis	1		1		1	1
**TGF-beta signaling pathway**	1		1		1	1
B cell receptor signaling pathway	1		1		1	1
mRNA surveillance pathway	1	1	2			0

*Significance of association based on p value ≤ 0.05 and False Discovery Rate (FDR) correction. Pathways with p-values < 0.05 are shown which are significant. Numbers indicate number of vSMs or vSSs*.

## Author Contributions

AB and AM collected and analyzed the data and wrote the manuscript with RT.

### Conflict of Interest Statement

The authors declare that the research was conducted in the absence of any commercial or financial relationships that could be construed as a potential conflict of interest.

## Supplementary Material

Supplementary Table 1 Lists of all raw data.

Supplementary Table 2 Lists analysis of other RNA viruses that also show vSMs and vSSs.
